# Progression of White Matter Hyperintensities Contributes to Lacunar Infarction

**DOI:** 10.14336/AD.2017.0808

**Published:** 2018-06-01

**Authors:** Xin Xu, Yuanyuan Gao, Renyuan Liu, Lai Qian, Yan Chen, Xiaoying Wang, Yun Xu

**Affiliations:** ^1^Department of Neurology, Affiliated Drum Tower Hospital, and Jiangsu Key Laboratory for Molecular Medicine, Nanjing University Medical School, Nanjing 210008, China.; ^2^Jiangsu Province Stroke Center for Diagnosis and Therapy, Nanjing 210008, China.; ^3^Nanjing Neuropsychiatry Clinic Medical Center, Nanjing 210008, China.; ^4^Departments of Neurology, Massachusetts General Hospital, Harvard Medical School, Charlestown, MA, USA.

**Keywords:** progression of white matter hyperintensities, lacunar infarction, incidence, quantitative analysis

## Abstract

Both white matter hyperintensities (WMHs) and lacunar infarctions (LIs) are magnetic resonance imaging (MRI) markers of cerebral small vessel disease (SVD). However, the association between WMH and LI remains unclear. In this study, we asked whether WMH progression is related to LI occurrence using retrospective data. Overall, 8475 WMH patients with at least two MRI images were screened, and 187 patients were included in the final study; 76 patients had WMH with LI (WL), and 111 patients had WMH without LI (WOL). The 187 patients were divided into three groups according to WMH progression: Group 1 (no progression), Group 2 (0-53.64% WMH progression) and Group 3 (≥53.64% WMH progression). We found that both WMH volumes and Fazekas scores were higher in WL patients compared with those in WOL patients according to the 1^st^ and 2^nd^ MRI images (P<0.001), whereas WMH progression was not significantly different between these two groups (P>0.05). Importantly, we found that the occurrence rates for LI were increased in Groups 2 and 3 compared with those in Group 1. Multiple logistic regression analysis demonstrated that the risk of LI occurrence was significantly increased in Group 2 versus that in Group 1 (odds ratio, 3.36; 95% CI, 1.48 to 7.67; P=0.004) after adjusting for the baseline patient characteristics and the interval between the two MRI scans. Additionally, with a stratification time of less than 24 months, the risk of LI occurrence was higher in Group 2 versus that in Group 1, after adjusting for baseline confounding factors (odds ratio, 3.68; 95% CI, 1.51 to 8.99; P=0.004). In conclusion, we found that WMH progression was significantly associated with LI occurrence, particularly within the first two years, and that this progression could serve as an independent indicator of LI development.

White matter hyperintensities (WMHs) and lacunar infarctions (LIs) are the primary MRI representations of cerebral small vessel disease (SVD). SVD accounts for up to 25% of stroke and 50% of dementia cases [1-4] and is characterized by stereotypical radiological changes in MRI, including WMHs, LI, lacunes, enlarged perivascular space, cerebral microbleeds and brain atrophy [5]. SVD is also associated with vascular risk factors, particularly hypertension [6]. The clinical spectrum for SVD ranges from asymptomatic disease, which is detectable by brain imaging in healthy individuals, to extensive WMH and lacunar infarcts in vascular dementia [7, 8]. Approximately 70% of people over 65 years of age present with varying degrees of WMH after undergoing MRI [9, 10].

WMHs from SVD are thought to be caused by chronic ischaemia attributable to arteriolosclerosis of medullary arteries [11]. By contrast, LI is an acute ischaemic lesion of SVD primarily caused by segmental arterial disorganization and is usually secondary to hypertension [1]. Both WMH and LI are associated with age and hypertension [12, 13], and because these two pathologies share similar risk factors, they are often observed together. Large WMH volumes are clinically important because they are associated with cognitive impairment, dementia, gait disturbances and depression [14, 15]. Although most LIs appear to be “silent,” meaning they are not accompanied by stroke-like symptoms, they are thought to be associated with subtle cognitive dysfunction [16, 17]. However, the relationship between WMH volume and LI remains unclear. In this study, we aimed to determine whether WMH progression is related to LI occurrence.

## MATERIALS AND METHODS

### Study population

**Figure 1.**
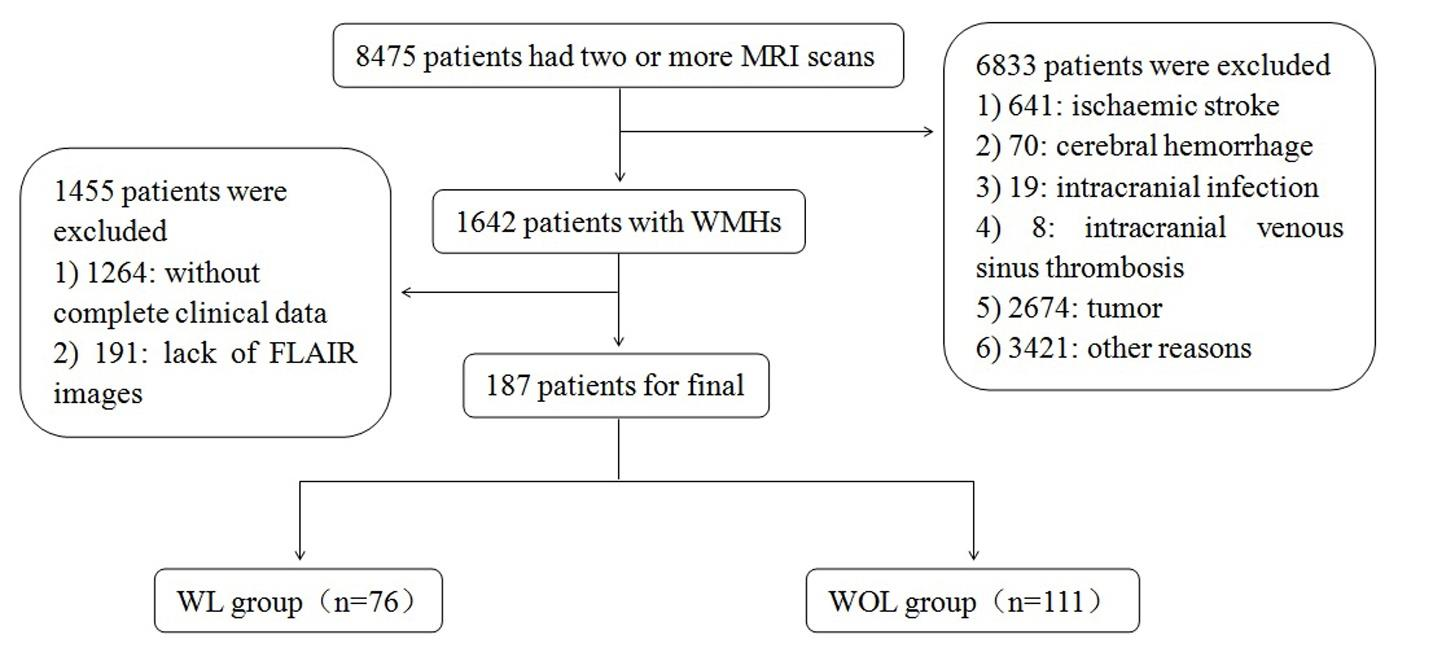
Diagrammatic sketch of the screening process.

A total of 8475 patients with WMHs on two or more MRI scans were studied retrospectively from January 1, 2011 to December 1, 2016 from the Drum Tower hospital imaging register centre of Nanjing University Medical School. Patients were recruited if they had WMHs on FLAIR images from their first MRI and had complete clinical data, including demographic data, a medical history, a physical examination and a laboratory examination. Exclusion criteria included cerebral infarction, cerebral haemorrhage, subarachnoid haemorrhage, intracranial and extracranial vascular lesions (Narrow degree over 50%), cerebral venous sinus thrombosis, central nervous system infections, leukoencephalopathy of nonvascular origin (immunological, demyelinating, metabolic, toxic, infection or other) or severe unrelated neurological diseases (e.g., cardiac, hepatic, or renal failure, cancer or other relevant systemic diseases). Consequently, 8288 patients were ruled out with 1264 patients lacking complete clinical data, 641 with ischaemic stroke, 70 with cerebral haemorrhage, 19 with intracranial infection, 8 with intracranial venous sinus thrombosis, 2674 with tumours, 191 patients lacking FLAIR images, and 3421 patients for other reasons. In the end, 187 patients were analysed in this study, including 76 patients with LI (WL) and 111 without LI (WOL) (Fig. 1). The Affiliated Drum Tower Hospital of Nanjing University Medical School Ethics Committee approved this study. The procedures were conducted according to institutional guidelines.

**Figure 2.**
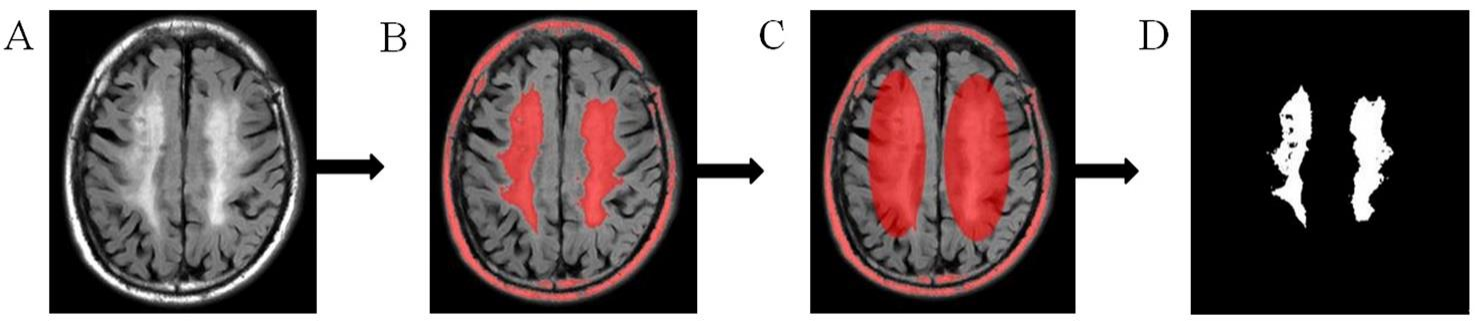
Quantitative steps of WMH volume. (A) Original FLAIR image. (B) The high signal area. (C) A sketch of the effective WMH area. (D) Extract of the effective WMH area.

### Risk factors

To assess relevant risk factors at baseline, we determined patient demographics (age, sex and medical history), history of hypertension, history of diabetes mellitus, clinical history of stroke, atrial fibrillation and myocardial infarction, history of dyslipidaemia, and past or present cigarette or alcohol use. Patients underwent a physical examination, systolic and diastolic blood pressure were measured, and laboratory examinations were performed, including tests for glucose (random blood glucose, fasting blood glucose, postprandial blood glucose and glycosylated haemoglobin), total cholesterol, high-density lipoprotein, triglycerides, low-density lipoprotein, apolipoprotein A-I, urea nitrogen, creatinine, uric acid and C-reactive protein.

### MRI examinations

MRI scanning was performed on a 1.5T or 3.0T scanner. The scanning protocol included a whole brain T1 magnetization-prepared rapid gradient echo sequence, a transversal T2-weighted turbo spin echo sequence, and a fluid-attenuated inversion recovery (FLAIR) pulse sequences.

### Radiological diagnosis of WMHs and LI

WMHs are hyperintense on T2-weighted or FLAIR sequences, and they can appear as isointense or hypointense (although they are not as hypointense as CSF) on T1-weighted sequences, depending on the sequence parameters and severity of the pathological changes [5]. White matter lesions, characterized by bilateral and mostly symmetrical hyperintensities on T2-weighted MRI, are common in older individuals.

LI is a small subcortical infarct with a diameter ranging from 3-15 mm on axial sections [5].

### Quantitative analysis of WMH volume

WMH volumes were quantified using the software programmes MRICRON (University of Nottingham School of Psychology, Nottingham, UK; www.mricro.com) and ITK-SNAP (University of Pennsylvania, Philadelphia, USA; www.itksnap.org) (Fig. 2). All scans were checked by visual inspection. First, we used the MRICRON software to extract the effective WMH area, and then we used the ITK-SNAP software to calculate the volume. To eliminate individual differences, WMH volume was standardized by intracranial volume (Standardized WMH volume = actual calculated WMH volume/intracranial volume × 100%). As baseline WMH volume is a strong predictor of WMH progression, we calculated WMH progression as a percentage of the baseline WMH volume using the formula WMH progression = (WMH2-WMH1)/WMH1*100 [15, 18]. Based on the state of WMH progression, patients were divided into three groups: no WMH progression group (Group 1) and a WMH progression group segmented into two subgroups (Group 2 and Group 3) according to the median measurement (non-normal distribution).

### Fazekas scale score

The degree of WMH severity was rated visually on axial FLAIR images using the modified Fazekas scale [19, 20], which is the most widely used and validated system for describing WMH severity. The scale divides WMHs into periventricular and deep, and periventricular WMHs were graded according to the following patterns: 0 = absent; 1 = caps or pencil-thin lining; 2 = smooth halo; and 3 = irregular periventricular WMH extending into a deep WMH. Deep WMHs were graded according to the following patterns: 0 = absent; 1 = punctate foci; 2 = beginning confluence of foci; and 3 = large fused areas. A total score (0 to 6) was acquired by adding the periventricular and deep WMH scores, and the results were compared using quantitative analyses.

### Statistical analysis

Data for the baseline characteristics were calculated separately for the two groups with and without lacunar infarction. Mean (standard deviation) or median (interquartile ranges) measurements were chosen depending on whether continuous variables had a normal distribution. Frequencies were used to describe categorical variables. Differences between groups were tested using an independent Student’s t-test for normally distributed continuous variables, with a Wilcoxon rank-sum test for variables that followed a skewed distribution, and a chi-squared test for categorical variables. Univariate and multivariate logistic regression analyses were used to assess the association between WMH progression and LI. The ORs and 95% CIs of WMH progression were calculated with no WMH progression as a reference. Potential covariates, such as age, gender, hypertension, the interval between the two MRI scans and others, were adjusted in the multivariate model. All P values were two-tailed, and P < 0.05 was considered statistically significant. Statistical analyses were performed using the SAS statistical package (version 9.3).

**Table 1:** Baseline characteristics.

	WL (n=76)	WOL (n=111)	P Value
Age, mean ± SD	72.45±10.93	68.37±10.81	0.013 *
Men, n (%)	52 (68.42)	61 (54.95)	0.064
Smoking, n (%)	12 (15.79)	13 (11.71)	0.421
Alcohol, n (%)	4 (5.26)	7 (6.31)	0.766
SBP, mean ± SD	139.33±19.19	131.47±14.91	0.002 **
DBP, mean ± SD	76.36±12.44	73.77±11.59	0.147
FPG, median (IQR)	5.27 (4.69-6.74)	5.21 (4.60-6.39)	0.391
RBG, median (IQR)	7.05 (5.60-9.00)	7.20 (6.00-8.90)	0.577
PBG, median (IQR)	10.40 (7.65-12.85)	8.00 (7.00-11.00)	0.005 **
HbA1c, median (IQR)	6.00 (5.60-6.70)	6.00 (5.50-6.70)	0.993
TC, median (IQR)	3.81 (3.14-4.67)	4.12 (3.58-4.90)	0.027 *
TG, median (IQR)	1.33 (0.86-1.89)	1.34 (0.95-1.96)	0.414
LDL, median (IQR)	1.96 (1.35-2.57)	2.21 (1.74-2.72)	0.121
HDL, median (IQR)	1.02 (0.89-1.15)	1.05 (0.86-1.25)	0.337
ApoA I, median (IQR)	1.06 (0.95-1.27)	1.15 (1.04-1.38)	0.009 **
BUN, median (IQR)	5.40 (4.40-6.96)	5.20 (4.30-6.30)	0.347
Cr, median (IQR)	70.50 (61.50-89.50)	66.00 (56.00-78.00)	0.044 *
UA, median (IQR)	315.5 (243.5-380)	315 (268-384)	0.412
CRP, median (IQR)	4.1 (3.0-10.3)	3.7 (2.5-4.8)	0.004 **
Hypertension, n (%)	37 (48.68)	34 (30.63)	0.013 *
Diabetes mellitus, n (%)	44 (57.89)	48 (43.24)	0.049 *
Dyslipidaemia, n (%)	46 (60.53)	60 (54.05)	0.380
Interval time, n (%)	19 (11-29)	15.5 (10-27)	0.2931

WMH = White matter hyperintensities; LI = Lacunar infarction; WL = WMH with LI; WOL = WMH with no lacunar infarction; SBP = Systolic blood pressure; DBP = Diastolic blood pressure; FPG = Fasting blood glucose; RBG = Random blood glucose; PBG = Postprandial blood glucose; HbA1c = Glycosylated haemoglobin; TC = Total cholesterol; TG = Triglyceride; LDL = Low density lipoprotein; HDL = High density lipoprotein; ApoA I = Apolipoprotein A I; BUN = Blood urea nitrogen; Cr = Creatinine; UA = Uric acid; CRP = C-reactive protein.

* Significant difference (P < 0.05);

** Significant difference (P<0.01).

## RESULTS

### Baseline characteristics

As shown in Table 1, multiple variables were different between the WL and WOL groups, including age, systolic blood pressure (SBP), postprandial blood glucose (PBG), total cholesterol (TC), apolipoprotein A-I (ApoA I), creatinine (Cr), C-reactive protein (CRP), diabetes mellitus and hypertension. Most of those variables were higher in the WL group (P<0.05), although TC and ApoA I were reduced relative to the WOL groups (P<0.05).

### WMH volume and Fazekas scale scores were different between WL and WOL groups

**Figure 3.**
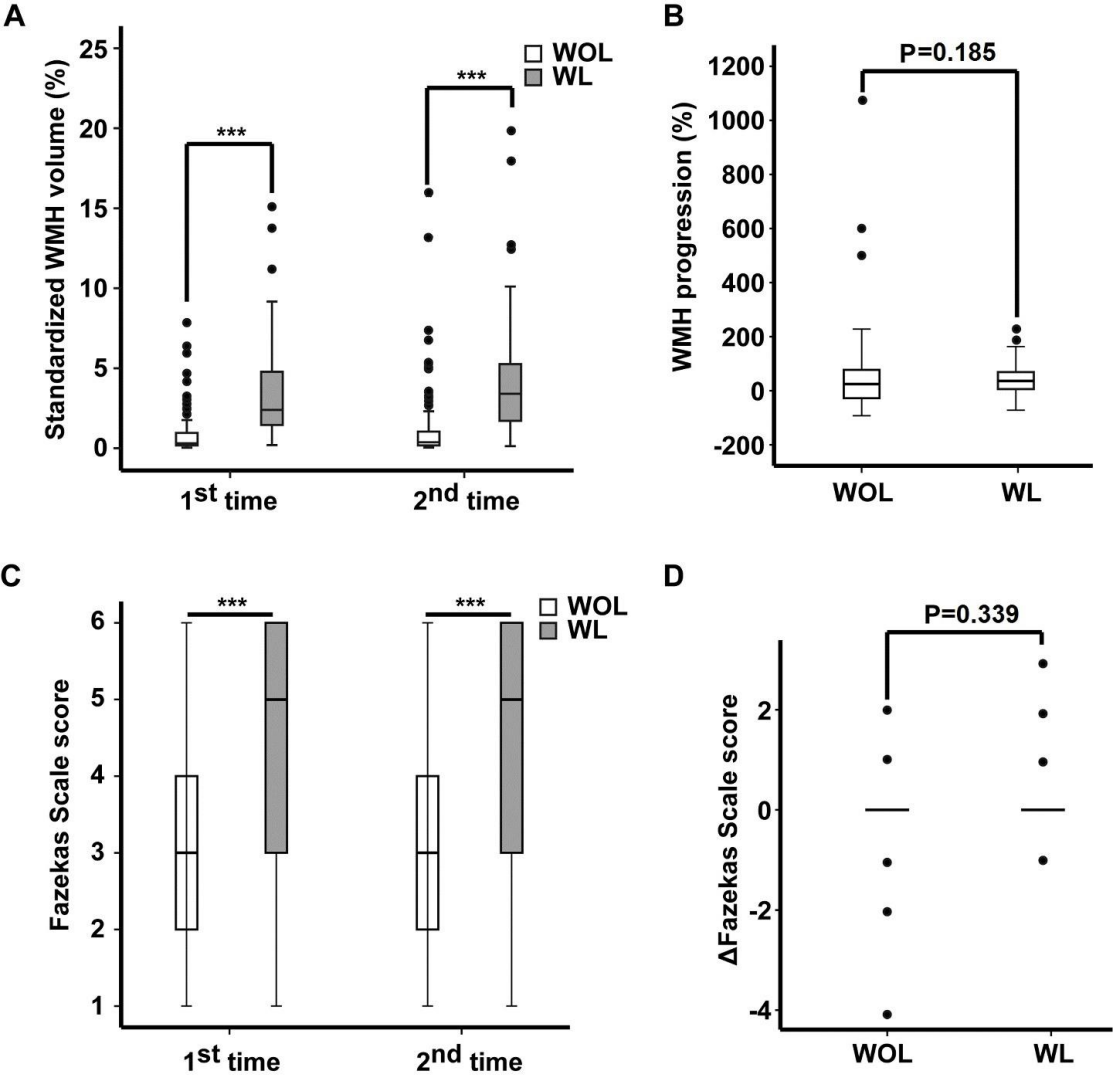
WMH volume and Fazekas scale scores for the 1^st^ and 2^nd^ MRI scans for WL and WOL. (A) Comparison of WMH volumes between two groups for two MRI scans. (B) Comparison of WMH progression between two groups. (C) Comparison of Fazekas scale score between two groups for two MRI scans. (D) Comparison of Fazekas scale score changes between two groups. ΔFazekas scale score = the total Fazekas scale score at 2^nd^ MRI scan - the total Fazekas scale score at 1^st^ MRI scan. *** Significant difference (P<0.001).

Based on MRI data from the 1^st^ and 2^nd^ scans, WMH volumes were significantly higher in WL compared with WOL (P<0.001, Fig. 3A). However, there were no significant differences in the progression of WMH volumes between WL and WOL (P = 0.185, Fig. 3B). Similarly, Fazekas scale scores of WMH showed the same statistical trends (Fig. 3C and D).

### LI occurred more frequently in patients with WMH progression

**Figure 4.**
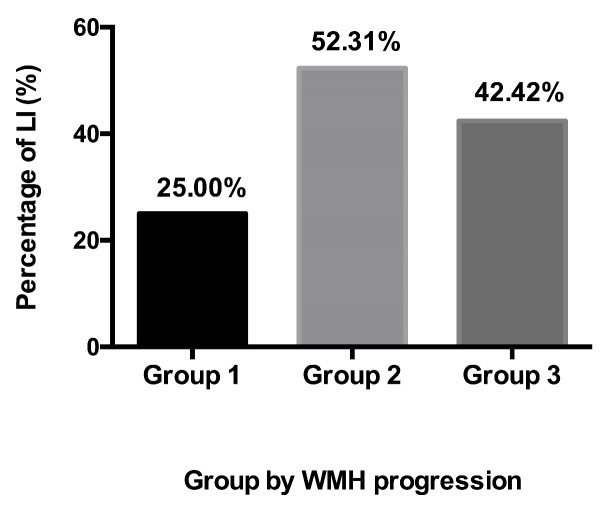
Prevalence of LI in patients stratified by WMH progression.

Next, we studied the distributions of LI occurrence in groups with different WMH progression. As shown in Figure 4, 25% of patients had LI in Group 1, whereas 52.3% and 42.4% of patients had LI in Groups 2 and 3, respectively (Fig. 4).

**Table 2:** Relationship between WMH Progression and LI.

Group by WMH Progression	Univariate Analysis		Multivariate Analysis	
	OR (95%CI)	P Value	OR (95% CI)^#^	P Value
Group 1(≤0)	1.00		1.000	
Group 2 (0, 53.64%)	3.29 (1.51-7.15)	0.003	3.36 (1.48-7.67)	0.004
Group 3 (≥53.64%)	2.21 (1.02-4.81)	0.046	2.14 (0.93-4.92)	0.073

WMH = White matter hyperintensity; OR = Odds Ratio.

# Odds ratios were adjusted for primary baseline characteristics of patients and the interval between the two MRI scans.

### Progression of WMH was associated with occurrence of LI

We next determined the association between WMH progression and LI occurrence using multiple logistic regression analysis. The univariate analysis showed that, compared with Group 1, the risks of LI occurrence were significantly increased in Group 2 (OR, 3.29; 95% CI, 1.51 to 7.15; P=0.003) and Group 3 (OR, 2.21; 95%CI, 1.02 to 4.81; P=0.046; Table 2). Furthermore, multivariate analysis showed that Group 2 patients still had a higher risk for LI occurrence than Group 1 patients after adjusting for age, sex, vascular history, the interval between the two MRI scans and other baseline confounding factors (OR, 3.36,95%CI, 1.48-7.67, P=0.004).

**Table 3:** WMH Progression stratified by time between the two times for MRI.

Group by WMH Progression	≤24 months		>24 months	
	OR(95%CI)	P Value	OR(95%CI)	P Value
Group 1(≤0)	1.00		1.000	
Group 2 (0, 53.64%)	3.68 (1.51-8.99)	0.004	2.40 (0.48-11.93)	0.285
Group 3 (≥53.64%)	2.39 (0.94-6.05)	0.066	1.83 (0.40-8.49)	0.438

WMH = White matter hyperintensity; OR = Odds Ratio.

All odds ratios were adjusted for age, sex, vascular history and other primary baseline confounding factors for LI.

In addition, we assessed whether the associations between WMH progression and LI occurrence were time-dependent by dividing patients into two groups: less than or more than 24 months. Multivariate analysis showed that the risk of LI occurrence was greater in Group 2 compared with that in Group 1 within 24 months, after controlling for age, sex and other main baseline confounding factors for LI (OR, 3.68; 95%CI, 1.51-8.99; P=0.004), whereas there was no significant correlation between WMH progression and LI beyond two years (Table 3).

### Discussion

The main findings of this study were as follows: 1) WMH patients with LI had more severe white matter lesions than patients without LI, according to WMH quantitative analysis and Fazekas scale score; and 2) WMH progression increased the risk of LI occurrence, particularly within two years.

WMH and LI have certain vascular risk factors in common, such as hypertension, diabetes mellitus, smoking and others [18, 21-23], which was supported by our baseline data. WL subjects had a significantly higher burden of age, SBP, PBG, CRP, diabetes mellitus, and hypertension compared with WOL subjects, whereas TC and ApoA I levels were lower in WL compared with those in WOL patients (P<0.05). However, when performing multiple logistic regression analysis, we found that these baseline confounding factors did not affect the final conclusions. As for the association between cholesterol levels and LI or WMH, current studies on this topic have conflicting results. Interestingly, one previous study reported that vascular risk factors, such as hypercholesterolemia, hyperlipidaemia and low body mass index seem to have protective effects against WMH progression [24], which is consistent with our results (Table 1).

One previous publication indicated that greater WMH burden was independently associated with larger volumes of small subcortical infarcts as well as poor 90-day outcomes after a small subcortical infarct [23]. LI frequently coexists with WMH, and both of these processes can lead to cognitive impairment [25]. It was reported that approximately 50% of LIs were located at the edge of a WMH [26]. Therefore, these studies suggest that WMH progression does increase the risk of LI, although how WMH burden predicts LI occurrence is poorly understood. For the first time, this study shows that WMH progression increases the risk of LI occurrence. Additionally, we found that the sub-median (0 to 53.64% WMH progression) rather than the super-median (over 53.64% WMH progression) group was associated with the occurrence and development of LI. Or in other words, LIs do not appear to develop when WMH progression has reached a certain load. These results could be interpreted to say that, from a pathological standpoint, decreased vascular density in WMH may decrease the incidence of acute infarctions [26]. On the other hand, it could be that some local LIs overlap with extensive WMH, which would lead to an underestimation of LI incidence.

Furthermore, we found that WMH leading to LI was time dependent and that the risk of LI occurrence was higher within the first 2 years post-WMH. Few longitudinal studies have described the progression of WMH and lacunes. The multicentre, multinational Leukoaraiosis and Disability study included a total of 396 patients who were followed for 3 years, and results showed that one in five subjects showed at least one new lacune within 3 years, and the new lacunes in a relatively high proportion of subjects were associated with stratification by WMH severity [18]. Moreover, Schmidt reported that of 273 participants (mean age 60 years), 17.9% individuals had WMH progression and 2.2% had a new LI within 3 years [27]. Therefore, it appears that early treatment of WMH progression would be beneficial.

Some studies have indicated that WMH is secondary to LI [22], whereas others have reported that WMH promotes the development of LI [28]. One potential mechanism of the former is that lacunar infarcts affect white matter tract integrity [22]. By contrast WMHs might promote LI by lowering cerebral blood flow (CBF) in the WMH [28]. Traylor M. and colleagues found that WMH-associated genetic variants also increased the risk of lacunar infarcts, and therefore, WMH and LI may share pathogeneses [29].

### Limitations

This study has several limitations. First, this was a single-centre, retrospective cohort research plan, which should not be extensively relied on in clinical practice. Second, although a large number of patients over six years of records were screened, only 187 patients met the inclusion criterion, which lowers the statistical power of this study. A future study involving multiple centres and more patients would help to determine the accuracy of these findings.

### Conclusions

We show that WMH progression was significantly associated with the occurrence of LI within a certain time frame and that WMH progression may serve as an independent indicator of LI occurrence. Our study suggests that WMH should be addressed at the early stages of evolution in clinical practice.
